# Grape Seed Proanthocyanidin Ameliorates FB_1_-Induced Meiotic Defects in Porcine Oocytes

**DOI:** 10.3390/toxins13120841

**Published:** 2021-11-25

**Authors:** Wenhui Li, Yijing He, Hongyu Zhao, Lei Peng, Jia Li, Rong Rui, Shiqiang Ju

**Affiliations:** College of Veterinary Medicine, Nanjing Agricultural University, Nanjing 210095, China; liwenhui202112@163.com (W.L.); heyijingnjau@163.com (Y.H.); zhaohongyunjau@163.com (H.Z.); pengleinjau@163.com (L.P.); lijia19960807@163.com (J.L.); rrui@njau.edu.cn (R.R.)

**Keywords:** porcine oocytes, fumonisin B_1_, grape seed proanthocyanidin, mitochondria, oxidative stress

## Abstract

Fumonisin B_1_ (FB_1_), as the most prevalent and toxic fumonisin, poses a health threat to humans and animals. The cytotoxicity of FB_1_ is closely related to oxidative stress and apoptosis. The purpose of this study is to explore whether Grape seed proanthocyanidin (GSP), a natural antioxidant, could alleviate the meiotic maturation defects of oocytes caused by FB_1_ exposure. Porcine cumulus oocyte complexes (COCs) were treated with 30 μM FB_1_ alone or cotreated with 100, 200 and 300 μM GSP during in vitro maturation for 44 h. The results show that 200 μM GSP cotreatment observably ameliorated the toxic effects of FB_1_ exposure, showing to be promoting first polar body extrusion and improving the subsequent cleavage rate and blastocyst development rate. Moreover, 200 μM GSP cotreatment restored cell cycle progression, reduced the proportion of aberrant spindles, improved actin distribution and protected mitochondrial function in FB_1_-exposed oocytes. Furthermore, reactive oxygen species (ROS) generation was significantly decreased and the mRNA levels of *CAT*, *SOD2* and *GSH-PX* were obviously increased in the 200 μM GSP cotreatment group. Notably, the incidence of early apoptosis and autophagy level were also significantly decreased after GSP cotreatment and the mRNA expression levels of *BAX*, *CASPASE3*, *LC3* and *ATG5* were markedly decreased, whereas BCL2 and mTOR were observably increased in the oocytes after GSP cotreatment. Together, these results indicate that GSP could exert significant preventive effects on FB_1_-induced oocyte defects by ameliorating oxidative stress through repairing mitochondrial dysfunction.

## 1. Introduction

Fumonisins are toxic secondary metabolites produced by Fusarium spp; they are mainly found in corn and corn-based products, but also in wheat, rice, barley, rye, oat, millet and other grain products [1]. Among the numerous fumonisins found to date, fumonisin B_1_ (FB_1_) is the most prevalent and toxic, posing detrimental hazards to animals and humans health [2]. A survey of fumonisin contamination in maize samples from eight provinces in China showed that a total of 67.1% of all maize samples were contaminated with FBs, with the average concentrations of FB_1_ being highest [3]. FB_1_ produces pleiotropic toxicities in animals, including hepatotoxicity [4], nephrotoxicity [5], neurotoxicity [6], immunotoxicity [7] and carcinogenicity [8]. The toxic mechanism of FB_1_ is related to oxidative stress, apoptosis and autophagy [9,10]. In recent years, the reproductive toxicity of FB_1_ has also caused great attention [11]. FB_1_ reportedly produces developmental toxicity in animal embryogenesis and maternal FB_1_ toxicity could even adversely affect the embryonic or fetal development, in turn leading to mortality [12,13]. Dietary FB_1_ could exert significant negative effects on sperm production and semen quality in rabbits [14] and boars [15]. Moreover, exposure to FB_1_ has adverse effects on granulosa cell proliferation and steroid production in pigs [16].

Mammalian oocyte meiosis represents a specialized cell cycle that consists of two consecutive M phases, without intervening S phase. During meiosis, the precise cytoskeleton dynamic distribution and correct cell cycle progression are crucial for the successful maturation of oocytes [17,18]. As the abundant organelles and the main source of reactive oxygen species (ROS) production, mitochondria play vital roles in energy production and cell metabolism [19]. When the function of mitochondria is impaired, the permeability of the inner mitochondrial membrane increases and excessive ROS are produced, leading to oxidative stress and autophagy in oocytes [18,20,21]. Our previous study confirmed that 30 µM FB_1_ exerts toxic effects on the meiotic maturation of porcine oocytes by destroying spindle dynamics and blocking cell cycle progression and its cytotoxicity was related to mitochondrial dysfunction-induced oxidative stress and apoptosis [22]. However, little information is available on the approaches to alleviate FB_1_ toxicity.

Proanthocyanidins (PACs), also known as condensed tannins, are one type of active phenolic group with natural antioxidant qualities and exist in a wide variety of plants, including fruits, seeds, flowers, nuts and bark [23]. Grape seeds are a rich source of PACs [24]. Grape seed proanthocyanidins (GSPs) have many advantages, such as low cost, high bioavailability and rapid absorption upon oral administration [25]. Accumulating evidence has shown that GSP, a natural antioxidant, exhibits powerful free radical scavenging ability and superior performance to vitamin C, vitamin E and β-carotene and is widely used for antioxidation or antiapoptosis [26,27]. Dietary supplementation with GSP can not only alleviate aflatoxin B_1_-induced oxidative stress by decreasing lipid peroxidation and enhancing the activity of antioxidant-related enzymes, but can also suppress excessive apoptosis by regulating the mitochondrial-mediated pathway in the spleen and bursa of Fabricius of broilers [28]. Regarding the reproductive system, GSP can antagonize the testicular toxicity caused by cisplatin [29], nickel sulfate [30], cadmium (Cd) [31] and diethylhexyl phthalate (DEHP) [32] in a rat model. Furthermore, grape seed proanthocyanidin extract (GSPE) could effectively prevent azathioprine-induced fetal malformations in rats and the ovarian aging process by reducing oxidative stress in hens [33,34]. Moreover, grape seed procyanidin B2 (GSPB2) exerted positive effects on reducing oxidative stress-induced granulosa cell apoptosis [35]. Although previous studies have indicated the beneficial effects of GSP on reproductive toxicity induced by different factors, it is unclear whether GSP has protective effects on the toxicity of FB_1_ exposure in porcine oocytes.

In the present study, porcine oocytes were used to investigate the possible protective effects of GSP on FB_1_-exposed oocyte maturation in vitro. Our results indicate that GSP could abrogate FB_1_-mediated toxicity of meiotic defects in porcine oocytes by ameliorating oxidative stress through alleviating mitochondrial dysfunction.

## 2. Results

### 2.1. GSP Ameliorated Meiotic Maturation Defects in FB_1_-Exposed Porcine Oocytes

**Figure 1:**
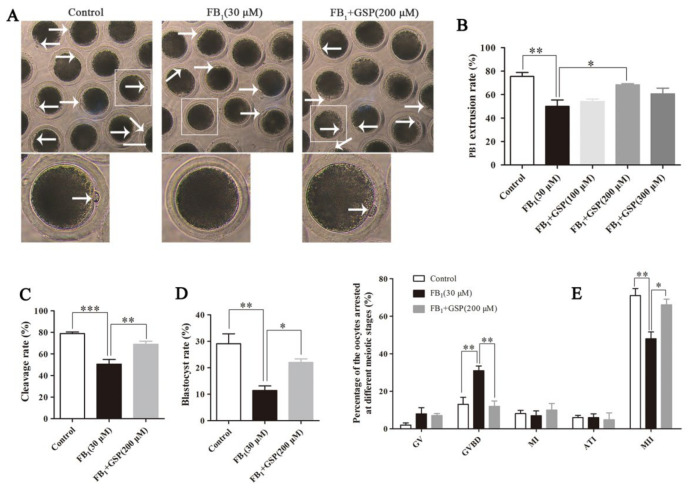
Effects of GSP on meiotic maturation and embryo developmental potential of FB_1_-exposed oocytes. (**A**) Morphology of oocytes of the control, 30 μM FB_1_-exposed and FB_1_+200 μM GSP cotreatment groups after having been cultured for 44 h. Scale bar, 100 μm; arrow, the first polar body (PB1). (**B**) Effects of gradient concentrations of GSP on the PB1 extrusion rate of the 30 μM FB_1_-exposed oocytes (*n* = 98 for control group; *n* = 98 for FB_1_ group; *n* = 100 for FB_1_+ 100 μM GSP group; *n* = 98 for FB_1_+ 200 μM GSP group; *n* = 99 for FB_1_+ 100 μM GSP group). (**C**,**D**) Cleavage rate and blastocyst rate in oocytes from the control (*n* = 110), 30 μM FB_1_-exposed (*n* = 112) and FB_1_+200 μM GSP cotreatment (*n* = 108) groups at 48 h and 168 h post-activation. (**E**) The percentage of oocytes arrested at different meiotic stages after 44 h of culture in vitro (*n* = 99 for control group; *n* = 100 for FB_1_ group; *n* = 100 for FB_1_+ 200 μM GSP group). Three replicates were performed and results are expressed as the mean ± standard error (SE). The letter “*n*” means the total number of oocytes in each group of 3 independent replicates. Significant difference: * *p* < 0.05 was considered significant; ** *p* < 0.01 was considered highly significant; *** *p* < 0.001 was considered very significant. FB_1_, Fumonisin B_1_; GSP, Grape seed proanthocyanidin; GV, germinal vesicle; GVBD, germinal vesicle breakdown; MI, metaphase I; ATI, anaphase–telophase I; MII, metaphase II.

As shown in Figure 1A,B, 30 μM FB_1_ exposure markedly decreased the rate of the first polar body (PB1) extrusion to 49.96 ± 4.75% (*p* < 0.01) compared to that in the control group (75.42 ± 3.07%). However, 200 μM GSP cotreatment significantly increased the PB1 extrusion rate of FB_1_-exposed oocytes (68.38 ± 0.76% vs. 49.96 ± 4.75%, *p* < 0.05, Figure 1B) and showed no differences compared to the control group. These results indicate that FB_1_ exposure caused the failure of oocyte meiotic maturation and that GSP had a protective effect against FB_1_-induced meiotic defects. According to the results, a concentration of 200 μM GSP was used in further experiments.

We further detected the embryo development potential. As shown in Figure 1C, after 48 h of embryo culture, the cleavage rate in the FB_1_-exposed group (50.50 ± 3.85%) was significantly lower than that in the GSP cotreatment group (68.93 ± 2.57%, *p* < 0.01) and extremely significantly lower than that in the control group (78.91 ± 1.24%, *p* < 0.001), but there was no significant difference between the GSP cotreatment group and the control group. As expected, after 168 h of embryo culture, the quantification analysis of the blastocyst rate showed a similar trend to the cleavage rate. As shown in Figure 1D, the blastocyst rate of the FB_1_-exposed group was 11.45 ± 1.47%, which was observably lower than that of the control group (29.10 ± 3.26%, *p* < 0.01). Conversely, the blastocyst rate was significantly increased to 22.03 ± 1.14% (*p* < 0.05) after cotreatment with GSP. These results revealed that GSP improved the embryo development potential of FB_1_-exposed oocytes.

### 2.2. GSP Protected the Cell Cycle Progression of FB_1_-Exposed Porcine Oocytes

As shown in Figure 1E, the proportion of metaphase II (MII) stage oocytes in the control group (71.02 ± 3.22%) was prominently higher than that in the FB_1_-exposed group (48.00 ± 3.16%, *p* < 0.01). Additionally, only 13.08 ± 3.25% of oocytes were arrested at the germinal vesicle breakdown (GVBD) stage in the control group, while a higher percentage of FB_1_-exposed oocytes was arrested at the GVBD stage (31.00 ± 2.18%, *p* < 0.01), leading to maturation failure. However, after GSP cotreatment, the percentage of oocytes arrested at the GVBD stage was significantly reduced to 12.01 ± 2.45% (*p* < 0.01) and the proportion of MII stage oocytes was increased to 66.09 ± 2.62% (*p* < 0.05). These results demonstrate that GSP could salvage the harmful effects of FB_1_ on meiotic progression in porcine oocytes.

### 2.3. GSP Alleviated the Defects of Spindle Assembly and Actin Distribution in FB_1_-Exposed Oocytes

**Figure 2:**
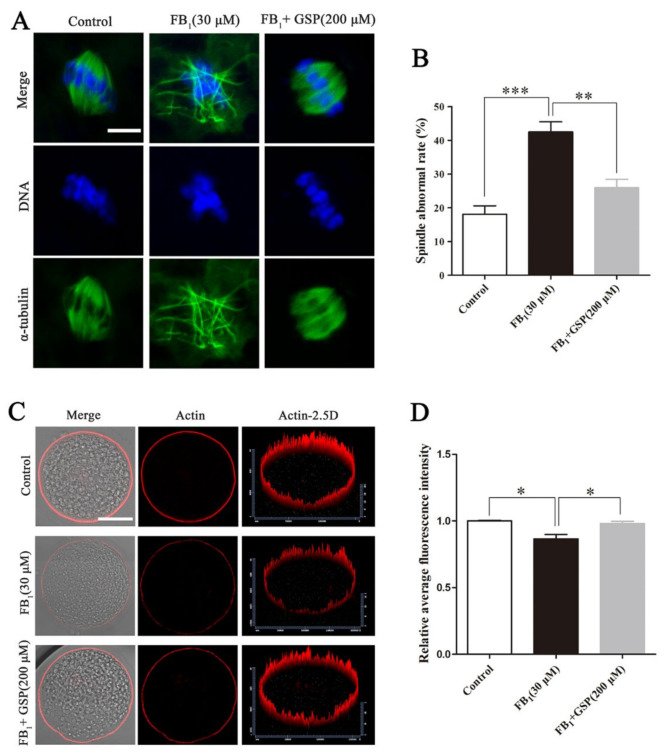
Effect of GSP on spindle assembly and actin distribution in FB_1_-exposed oocytes. (**A**) Typical fluorescent images of spindle morphology and chromosome alignment of oocytes in the control, 30 μM FB_1_-exposed and FB_1_+200 μM GSP cotreatment groups after cultured for 28 h. Scale bar, 10 μm; green, α-tubulin; blue, DNA. (**B**) Spindle abnormal rate in oocytes from the control (*n* = 99), 30 μM FB_1_-exposed (*n* = 101) and FB_1_+200 μM GSP cotreatment (*n* = 100) groups. (**C**) Typical fluorescent images of actin distribution in oocytes from the control, 30 μM FB_1_-exposed and FB_1_+200 μM GSP cotreatment groups. Scale bar, 50 μm; red, actin. (**D**) Relative average fluorescence intensity of actin in oocytes from the control (*n* = 59), 30 μM FB_1_-exposed (*n* = 59) and FB_1_+200 μM GSP cotreatment groups (*n* = 60). Three replicates were performed and results are expressed as the mean ± SE. The letter “*n*” means the total number of oocytes in each group of 3 independent replicates. Significant difference: * *p* < 0.05 was considered significant; ** *p* < 0.01 was considered highly significant; *** *p* < 0.001 was considered very significant.

As shown in Figure 2, most oocytes in the control group showed regular α-tubulin morphology and chromosome alignment along the metaphase plate to assemble into typical metaphase spindles. In contrast, the proportion of oocytes with aberrant α-tubulin and misaligned chromosomes in the FB_1_-exposed group (42.50 ± 2.59%) was prominently higher than that in the control group (18.13 ± 2.15%, *p* < 0.001, Figure 2B). However, GSP cotreatment observably reduced the proportion of FB_1_-exposed oocytes with spindle assembly defects to 25.98 ± 2.17 (*p* < 0.01). Moreover, as shown in Figure 2C,D, the analysis of relative average fluorescence intensity confirmed that the actin signals in FB_1_-exposed oocytes were strikingly decreased compared with those in the control group (0.866 ± 0.027 vs. 1.000 ± 0.004, *p* < 0.05), while the actin signals were significantly enhanced after GSP cotreatment (0.866 ± 0.027 vs. 0.980 ± 0.014, *p* < 0.05). There was no statistical difference in spindle assembly and actin distribution between the control and GSP cotreatment groups. These results demonstrate that GSP could rescue meiotic defects by protecting spindle structure and actin distribution in FB_1_-exposed oocytes.

### 2.4. GSP Protected Mitochondrial Function in FB_1_-Exposed Oocytes

**Figure 3:**
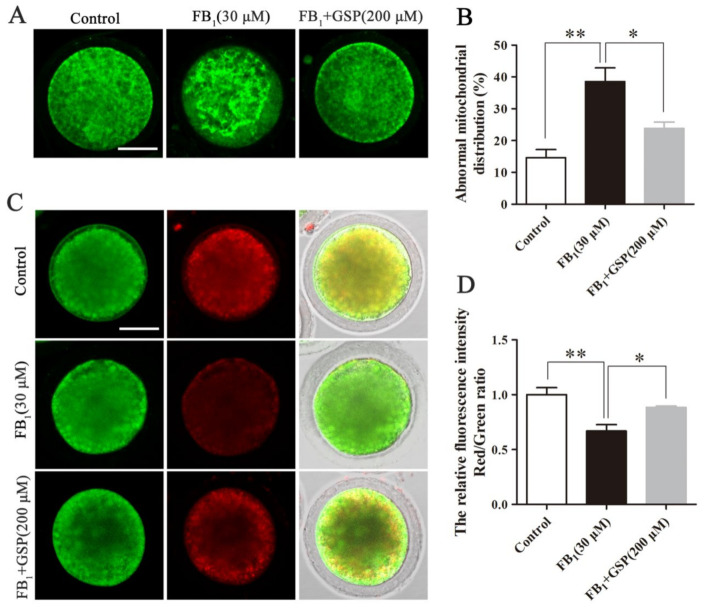
Effect of GSP on mitochondrial function in FB_1_-exposed oocytes. (**A**) Typical fluorescent images of mitochondrial distribution in oocytes from the control, 30 μM FB_1_-exposed and FB_1_+200 μM GSP cotreatment groups. Scale bar, 50 µm; green, MitoTracker Green. (**B**) The rate of oocytes that exhibited aberrant mitochondrial distribution in the control (*n* = 100), 30 μM FB_1_-exposed (*n* = 101) and FB_1_+200 μM GSP cotreatment groups (*n* = 100). (**C**) Typical fluorescent images of JC-1-stained oocytes from the control, 30 μM FB_1_-exposed and FB_1_+200 μM GSP cotreatment groups. Scale bar = 50 µm; green, JC-1 monomer; red, JC-1 aggregates. (**D**) The relative ratio of JC-1 red/green fluorescence signals in oocytes from the control, 30 μM FB_1_-exposed and FB_1_+200 μM GSP cotreatment groups (*n* = 100 for all groups). Three replicates were performed and results are expressed as the mean ± SE. The letter “*n*” means the total number of oocytes in each group of 3 independent replicates. Significant difference: * *p* < 0.05 was considered significant; ** *p* < 0.01 was considered highly significant.

As shown in Figure 3A,B, most control oocytes exhibited homogeneous mitochondrial distribution throughout the cytoplasm, while a higher percentage of FB_1_-exposed oocytes (38.58 ± 3.72%) displayed irregular clusters and inhomogeneous mitochondrial distribution compared with the control group (14.63 ± 2.22%, *p* < 0.01). However, the percentage of oocytes with abnormal mitochondrial distribution in the GSP cotreatment group was markedly decreased to 23.85 ± 1.69% (*p* < 0.05) compared with the FB_1_-exposed group. Furthermore, we also assessed the alterations of the mitochondrial membrane potential (MMP) by JC-1 staining and the results show that FB_1_ exposure significantly decreased the ratio of red to green fluorescence intensity compared with the control group (Figure 3C,D; 0.670 ± 0.049 vs. 1.000 ± 0.056, *p* < 0.01), indicating the decline of the MMP, while GSP cotreatment alleviated the trend of MMP decline in the FB_1_-exposed oocytes to some extent (0.670 ± 0.049 vs. 0.884 ± 0.009, *p* < 0.05). These data demonstrate that GSP could protect porcine oocytes from mitochondrial dysfunction with FB_1_ exposure.

### 2.5. GSP Alleviated Oxidative Stress in FB_1_-Exposed Oocytes

**Figure 4:**
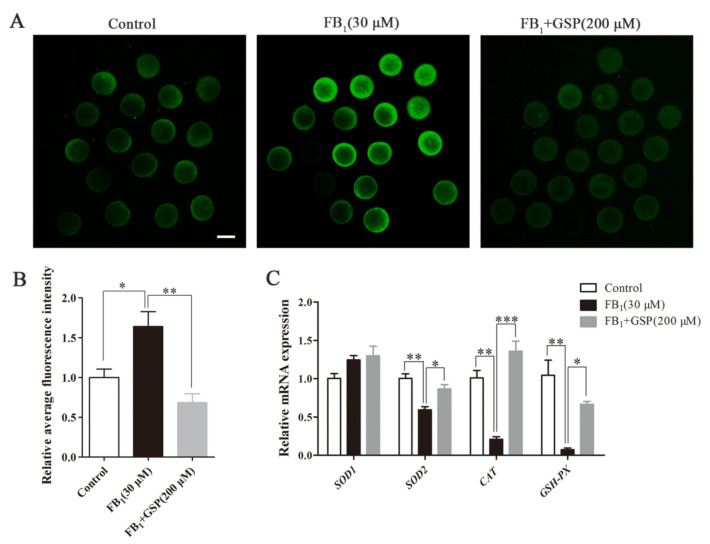
Effect of GSP on oxidative stress in FB_1_-treated porcine oocytes. (**A**) Typical fluorescent images of ROS-stained oocytes from the control, 30 μM FB_1_-exposed and FB_1_+200 μM GSP cotreatment groups. Scale bar, 100 μm; green, DCFHDA. (**B**) ROS levels in oocytes from the control, 30 μM FB_1_-exposed and FB_1_+200 μM GSP cotreatment groups (*n* = 100 for all groups). (**C**) The mRNA expression levels of *SOD1*, *SOD2*, *CAT* and *GSH-PX* genes in oocytes from the control, 30 μM FB_1_-exposed and FB_1_+200 μM GSP cotreatment groups. Three replicates were performed and results are expressed as the mean ± SE. The letter “*n*” means the total number of oocytes in each group of 3 independent replicates. Significant difference: * *p* < 0.05 was considered significant; ** *p* < 0.01 was considered highly significant; *** *p* < 0.001 was considered very significant.

As shown in Figure 4A,B, the relative fluorescence intensity analysis indicated that ROS signals were obviously increased in FB_1_-exposed oocytes compared with the control group (1.640 ± 0.163 vs. 1.000 ± 0.092, *p* < 0.05), while, following cotreatment of GSP, ROS generation was dramatically reduced (1.640 ± 0.163 vs. 0.684 ± 0.097, *p* < 0.01). Additionally, we further investigated whether FB_1_ exposure influenced the mRNA expression of antioxidant-related genes and significant decreases in *CAT*, *SOD2* and *GSH-PX* expression levels were observed in FB_1_-exposed oocytes compared with the control group (0.208 ± 0.027 for *CAT*, 0.681 ± 0.038 for *SOD2*, 0.072 ± 0.018 for *GSH-PX*; *p* < 0.01). On the other hand, GSP cotreatment upregulated the gene expressions of *CAT*, *SOD2* and *GSH-PX* (1.357 ± 0.109 for *CAT*, 0.905 ± 0.030 for *SOD2*, 0.666 ± 0.030 for *GSH-PX*; *p* < 0.05 for *SOD2* and *GSH-PX*, *p* < 0.001 for *CAT*; Figure 4C). There was no statistical difference in the gene expression of *SOD1* between the three groups. These data suggest that GSP alleviated oxidative stress in FB_1_-exposed oocytes by reducing ROS production and enhancing antioxidant enzyme activities.

### 2.6. GSP Reduced Early Apoptosis in FB_1_-Exposed Oocytes

**Figure 5:**
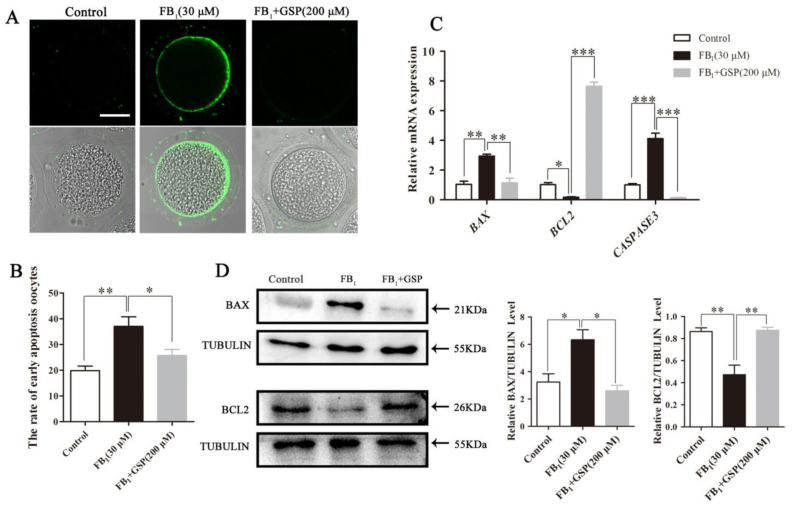
Effect of GSP on the early apoptosis in FB_1_-exposed oocytes. (**A**) Typical fluorescent images of Annexin-V-FITC-stained oocytes from the control, 30 μM FB_1_-exposed and FB_1_+200 μM GSP cotreatment groups. Scale bar, 50 μm; green, Annexin-V-FITC. (**B**) The rate of the oocytes that exhibited apoptotic positive signals in control (n = 101), 30 μM FB_1_-exposed (*n* = 100) and FB_1_+200 μM GSP cotreatment oocytes (*n* = 101). (**C**) The mRNA expression levels of *BAX*, *BCL2* and *CASPASE3* genes in oocytes from the control, 30 μM FB_1_-treated and FB_1_+200 μM GSP groups. (**D**) The protein expression of BAX and BCL2 in oocytes from the control, 30 μM FB_1_-exposed and FB_1_+200 μM GSP cotreatment groups. Three replicates were performed and results are expressed as the mean ± SE. The letter “*n*” means the total number of oocytes in each group of 3 independent replicates. Significant difference: * *p* < 0.05 was considered significant; ** *p* < 0.01 was considered highly significant; *** *p* < 0.001 was considered very significant.

As shown in Figure 5A,B, compared to the control group (19.85 ± 1.52%), 37.00 ± 3.28% of FB_1_-exposed oocytes exhibited a stronger Annexin-V signal (*p* < 0.01). However, after GSP cotreatment, the apoptotic rate of FB_1_-exposed oocytes was significantly reduced to 25.69±1.82% (*p* < 0.05). Additionally, relatively lower levels of *BAX* and *CASPASE3* and markedly higher levels of *BCL2* transcription were observed in the GSP cotreatment group than in the FB_1_-exposed group (2.944 ± 0.105 vs. 1.144 ± 0.261 for *BAX*, 4.124 ± 0.298 vs. 0.141 ± 0.010 for *CASPASE3*, 0.183 ± 0.027 vs. 7.650 ± 0.228 for *BCL2*; *p* < 0.01 for *BAX*, *p* < 0.001 for *CASPASE3* and *BCL2*; Figure 5C). We also evaluated the effect of GSP on the protein expression of BAX and BCL2 in FB_1_-exposed oocytes and the results show a relatively lower BAX (6.337 ± 0.599 vs. 2.602 ± 0.325, *p* < 0.05) and higher BCL2 (0.472 ± 0.071 vs. 0.875 ± 0.021, *p* < 0.01) expression pattern in the GSP cotreatment group (Figure 5D), which is in accord with the transcriptional findings above. These results suggest that GSP inhibited apoptosis in FB_1_-exposed oocytes by upregulating BCL2 and downregulating BAX.

### 2.7. GSP Decreased Autophagy Levels in FB_1_-Exposed Oocytes

**Figure 6:**
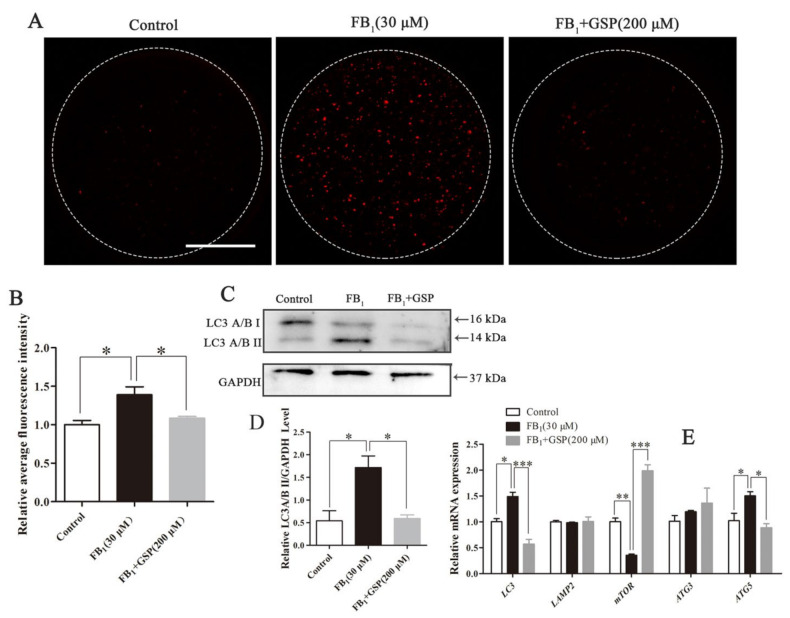
Effect of GSP on the autophagy of FB_1_-exposed oocytes. (**A**) Typical fluorescent images of LC3A/B-staining in oocytes from the control, 30 μM FB_1_-exposed and FB_1_+200 μM GSP cotreatment groups. Scale bar, 50 μm; red, LC3A/B. (B) Relative average fluorescence intensity of LC3A/B in oocytes from the control (*n* = 57), 30 μM FB_1_-exposed (*n* = 59) and FB_1_+200 μM GSP cotreatment groups (*n* = 58). (**C**) and (**D**) The protein expression of LC3A/B in oocytes from the control, 30 μM FB_1_-exposed and FB_1_+200 μM cotreatment GSP groups. (**E**) The mRNA expression levels of *LC3*, *LAMP*, *mTOR*, *ATG3* and *ATG5* genes in oocytes from the control, 30 μM FB_1_-exposed and FB_1_+200 μM GSP cotreatment groups. Three replicates were performed and results are expressed as the mean ± SE. The letter “*n*” means the total number of oocytes in each group of 3 independent replicates. Significant difference: * *p* < 0.05 was considered significant; ** *p* < 0.01 was considered highly significant; *** *p* < 0.001 was considered very significant.

As shown in Figure 6A,B, FB_1_-exposed oocytes exhibited more LC3A/B protein signal dots (1.392 ± 0.085) than control (1.000 ± 0.046, *p* < 0.05) and GSP cotreatment oocytes (1.083 ± 0.019, *p* < 0.05), indicating an increase in autophagy levels. Correspondingly, Western blot analysis confirmed an increase in LC3A/B II protein expression in FB_1_-exposed oocytes (1.718 ± 0.210 vs. 0.598 ± 0.137, *p* < 0.05), with a decrease in LC3A/B II expression after GSP cotreatment (1.718 ± 0.210 vs. 0.589 ± 0.067, *p* < 0.05; Figure 6C,D). Moreover, relatively lower levels of *LC3* (0.568 ± 0.075 vs. 1.489 ± 0.068, *p* < 0.001) and *ATG5* (0.887 ± 0.062 vs. 1.502 ± 0.065, *p* < 0.05) and significantly higher levels of *mTOR* (1.986 ± 0.095 vs. 0.354 ± 0.019, *p* < 0.001) transcription were observed in the GSP cotreatment group compared with the FB_1_-exposed group (Figure 6E). The gene expression of *LAMP2* and *ATG3* showed no differences among the three groups. These results suggest that GSP could reduce autophagy levels in FB_1_-exposed oocytes by upregulating *mTOR* and downregulating *LC3* and *ATG5*.

## 3. Discussion

FB_1_ is one of the most toxic fumonisins and has been shown to cause toxic effects in humans and animals [10]. Recently, the reproductive toxicity of FB_1_ has attracted great attention. Our previous studies reported the toxic effects of FB_1_ on porcine oocyte *IVM* and its cytotoxicity was associated with the induction of oxidative stress and apoptosis [22]. GSP is a powerful antioxidant and previous studies have demonstrated a broad spectrum of pharmacological and therapeutic benefits of GSP [24,27]. Herein, porcine oocytes were used as a research model to investigate the beneficial effects of GSP on ameliorating FB_1_-induced oocyte damage during meiotic maturation in vitro. Our results indicate that GSP administration protected oocytes from FB_1_-induced maturation defects by protecting cell cycle progression, protecting cytoskeletal structure and inhibiting apoptosis and autophagy. Moreover, the protective effects of GSP on FB_1_-treated oocytes were related to reducing oxidative stress by repairing FB_1_-induced mitochondrial dysfunction.

It has been reported that procyanidins to trimers can be absorbed from the digestive tract and are present in rat blood and urine after oral administration of GSPE [36]. Dietary GSPE could effectively prevent the azathioprine-induced ovarian aging process by reducing oxidative stress in hens [34] and intragastric administration of GSPB2 exerted positive effects on reducing oxidative stress-induced follicular granulosa cell apoptosis in mice [35]. These results suggest that GSPE can be absorbed from the digestive tract and reach the blood and ovaries, exerting its antioxidant effect. Herein, we demonstrated that GSP could alleviate porcine oocyte meiotic defects caused by FB_1_ exposure. PB1 extrusion is considered a marker event for oocyte maturation [37]. Our results show that 200 μM GSP cotreatment significantly increased the PB1 extrusion rate, cleavage rate and blastocyst rate, relieving the adverse effects of FB_1_ on oocyte meiotic maturation. However, further in vivo studies are required to verify the specific concentration of GSP reaching the oocyte in pig ovaries. This finding is in accord with a previous study showing that GSPE administration markedly alleviated Cd-induced embryo toxic effects by upregulating meiosis-related gene expression and restoring antioxidative levels [38]. Thus, we suggest that GSP is a potential candidate to alleviate porcine oocyte meiotic defects caused by FB_1_.

We further explored the reasons for the protective effects of GSP by detecting the meiotic progression and cytoskeleton dynamics of oocytes. A previous study showed that FB_1_ treatment impeded swine peripheral blood mononuclear cell proliferation and increased the percentage of cells blocked in G0/G1 phase of the cell cycle [39]. Another study in human umbilical vein endothelial cells verified that FB_1_ could adversely affect the cellular migration and cytoskeletal structure [40]. Our results indicate that FB_1_ disrupted spindle organization and actin distribution during the GVBD-MI transition in porcine oocytes and the failure of oocyte maturation might be caused by the dysfunctional cytoskeleton dynamics induced by FB_1_ exposure. Notably, GSP cotreatment restored cell cycle progression by promoting the successful GVBD-MI transition, rescued the stability of spindles and increased the actin distribution in FB_1_-exposed oocytes. Similarly, proanthocyanidin administration exerted effective effects on cisplatin-induced cycle arrest in renal cells and reorganized the actin cytoskeleton in endothelial cells [41,42]. Therefore, GSP might ameliorate cytoskeletal organization to rescue meiotic progression and promote FB_1_-exposed oocyte maturation.

Mitochondria, as abundant organelles in oocytes, are essential for oocyte cytoplasmic maturation [19]. Mitochondrial distribution and MMP can be used as two indexes to evaluate mitochondrial activity, which is related to the developmental potential of oocytes [43]. Studies have found that FB_1_ causes cytotoxicity through the mitochondrial signaling pathway [44]. An increased abnormal rate of mitochondrial distribution and decreased MMP were observed in the oocytes, suggesting that FB_1_ exposure caused mitochondrial dysfunction [45,46]. However, GSP administration alleviated mitochondrial defects in FB_1_-exposed oocytes to a certain extent. This result was also in keeping with the opinion that the bioactivity of GSP was linked to regulating mitochondrial function [47,48,49]. Therefore, repairing mitochondrial dysfunction might be an important way for GSP to rescue the meiotic maturation of FB_1_-exposed oocytes.

Mitochondrial dysfunction is frequently associated with ROS overproduction and excessive ROS production causes oxidative damage and early apoptosis in cells [50]. Increased ROS levels were associated with meiotic cell cycle arrest and cytoskeleton disorganization [51,52]. Based on our results regarding the dysfunctional mitochondria and meiotic injury caused by FB_1_ exposure, we next examined oxidative stress in FB_1_-exposed oocytes. The results show that FB_1_ treatment dramatically increased ROS generation in oocytes, in turn inducing oxidative stress, which might be due to the significant decrease in antioxidant gene (*SOD2*, *CAT*, *GSH-PX*) mRNA levels. These results are in keeping with previous studies that showed that the cytotoxicity of FB_1_ was associated with oxidative stress [10,53]. Moreover, we observed that GSP administration, as a natural antioxidant, could eliminate excessive ROS by upregulating the mRNA expression of *SOD2*, *CAT* and *GSH-PX*. However, there was no significant difference in the gene expression of *SOD1,* which indicates the specificity of FB_1_ and GSP towards *SOD2.* The ability of GSP to fight oxidative stress has been confirmed in other cell lines [28,33,47]. Taken together, these results suggest that GSP could prevent oxidative stress and the following meiotic defects by rescuing mitochondrial dysfunction in FB_1_-exposed oocytes.

Oxidative stress might exert negative effects on oocyte physiology by inducing apoptosis [52]. The oxidative stress-induced mitochondria-mediated pathway plays a crucial role in oocyte apoptosis [52,54]. We found that FB_1_ exposure induced apoptosis of the oocytes. Additionally, a decline in MMP, as an indicator of early cell apoptosis [20], was also observed in FB_1_-exposed oocytes. These results support previous findings in that the toxicity induced by FB_1_ is associated with apoptosis [9,10,55]. Intriguingly, our data further reveal that GSP cotreatment rescued the early apoptosis triggered by FB_1_. A study in lymphocytes reported that GSPE could reduce apoptosis induced by extracellular histones by upregulating BCL2 expression [56]. Collectively, our results indicate that the suppressive effects of GSP on apoptosis might be another mechanism against FB_1_ toxicity in oocytes.

The overproduction of ROS is related to the induction of autophagy [57]. Autophagy, a mechanism that acts as the bulk degradation of proteins and organelles [58], plays a vital role in various physiological and pathological contexts [59]. The opinion that excessive or prolonged autophagy induces cytotoxicity has been widely accepted [60]. Our data suggest that FB_1_ induced autophagy in porcine oocytes, which was further confirmed by the increased expression of autophagy-related genes (LC3 and ATG5) and proteins (LC3A/B II). Previous studies have shown that FB_1_ causes colonic damage through oxidative stress-associated apoptosis and autophagy in a murine model [55] and induced autophagy-mediated cell death in MARC-145 kidney cells [61]. Therefore, these data strongly suggest that FB_1_-induced autophagy is another factor contributing to the maturation failure of porcine oocytes. Alternatively, GSP administration markedly reduced autophagy in FB_1_-exposed oocytes by downregulating the expression of *LC3* and *ATG5* and upregulating the expression of *mTOR*. This effect of procyanidins modulating autophagy has been proposed in other models. A study reported that procyanidin could reduce the autophagy induced by influenza A virus by inhibiting the accumulation of LC3II [62]. Our results indicate that the protective effects of GSP on FB_1_-exposed oocytes might be related to the weakening of autophagy-induced cytotoxicity.

## 4. Conclusions

In summary, our data manifest that FB_1_ exposure disrupted oocyte maturation by impeding meiotic progression, disrupting cytoskeletal integrity and impairing mitochondrial function. In contrast, GSP cotreatment effectively antagonized the FB_1_ mediated toxicity to oocytes and its protective effect was related to reducing oxidative stress by repairing mitochondrial dysfunction. These data will improve our knowledge of the protective effect of GSP against FB_1_-induced oocyte toxic damage and provide new strategies for effective control of other reproductive toxicities of FB_1_.

## 5. Materials and Methods

### 5.1. Antibodies and Chemicals

FB_1_ (purity > 98%) was purchased from Sigma-Aldrich (St. Louis, MO, USA). GSP (purity ≥ 95%) was purchased from JF-NATURAL (Tianjin, China). Antibodies against α-tubulin (Abcam, London, UK), GAPDH (Cell Signaling Technology, Danvers, MA, USA), BCL2 (Proteintech Group, Chicago, USA), BAX (Bioss, Beijing, China), LC3 A/B (Cell Signaling Technology, Danvers, MA, USA), TRITC-labeled phalloidin (YEASEN, Shanghai, China), TRITC-labeled goat anti-rabbit IgG H+L (HUABio, Hangzhou, China) and HRP-labeled goat anti-rabbit IgG (H+L) (Beyotime Biotechnology, Shanghai, China) were purchased. MitoTracker Green fluorescent probe, JC-1 fluorescent probe and DCFHDA probe were purchased from Beyotime Biotechnology (Shanghai, China). Annexin-V-FITC fluorescent probe was purchased from Vazyme (Jiangsu, China).

### 5.2. Ethics Statement and Porcine Oocyte In Vitro Maturation

The experimental protocols were conducted according to the requirements of the Animal Research Ethics Committee of Nanjing Agricultural University (Permit Number: IACUC2020132, date of approval 4 March 2020.), China. Porcine oocytes collection and in vitro maturation were performed as previously described [63]. Briefly, porcine ovaries were obtained from a local slaughterhouse (SU SHI Meat Co., Ltd, Nanjing, China) and transported to the laboratory within 2 h. All oocytes in each experiment were from the same batch of ovaries. The COCs from 3 to 6 mm diameter follicles were collected and a group of approximately 25 COCs were selected and transferred into TCM-199 medium supplemented with 3.05 mM D-glucose, 0.57 mM L-cysteine, 0.91 mM sodium pyruvate, 10 IU/mL PMSG and hCG, 10 ng/mL of EGF, 0.1% (w/v) polyvinyl alcohol, 10% (*v/v*) porcine follicular fluid, 7.5 mg/mL of penicillin and 5.0 mg/mL of streptomycin for IVM at 38.5 °C and 5% CO_2_ with saturated humidity. After 28 or 44 h of culture, the time at which oocytes were supposed to reach metaphase I (MI) and MII stages [64], cumulus cells were removed with 0.1% (*w/v*) hyaluronidase. Eventually, the denuded oocytes were collected for subsequent experiments.

### 5.3. FB_1_ Exposure and GSP Treatment

Both FB_1_ and GSP were dissolved in dimethyl sulfoxide (DMSO) to 10 mM and 60 mM as stock solutions, respectively, and were stored at −20 °C. The stock solution of FB_1_ was diluted in IVM medium to produce a final concentration of 30 μM for FB_1_ treatment during in vitro oocyte culture as previously reported [22]. Based on previously reported results on effective protective concentrations of GSP in EA.hy926 cells, human epidermal keratinocytes and human granulosa cells [47,65,66], the GSP stock solution was diluted in IVM medium (supplemented with 30 μM FB_1_) to final concentrations of 100, 200 and 300 μM for the cotreatment of GSP with FB_1_ during IVM. The control group was supplemented with an identical concentration of DMSO.

### 5.4. Oocyte Parthenogenetic Activation and Early Embryo Culture In Vitro

Porcine oocyte parthenogenetic activation (PA) and embryo culture in vitro were performed as described in previous reports [67,68]. After washing three times in electric activation medium (1 mM CaCl_2_, 0.1 mM MgCl_2_, 0.3 M mannitol and 0.1% BSA), MII oocytes were activated using a CRY-3 Cell Fusion-Activation System (Xinzhi Biotechnology, Ningbo, China) under the following conditions: a single direct current pulse of 1.5 kV/cm for 80 µs. Then, oocytes were incubated for 4 h in a chemically assisted activation medium (PZM-3 supplemented with 2 mM 6-dimethylaminopurine and 5 μg/mL of cytochalasin B). Activated embryos were washed three times and cultured in PZM-3 supplemented with 0.4% (*w/v*) BSA at 38.5 °C and 5% CO_2_ with humidified air. The cleavage and blastocyst rates were examined under an inverted microscope (Olympus, Tokyo, Japan) at 48 h and 168 h post-activation [18,69].

### 5.5. Immunofluorescence Staining

The oocytes were fixed with 4% (*w/v*) paraformaldehyde (PFA) in phosphate buffered saline (PBS) at room temperature (RT) for 30 min, permeabilized with 1% (*w/v*) Triton X-100 in PBS at RT for 8 h and then blocked in 1% (*w/v*) BSA at RT for 1 h. For LC3A/B staining, the oocytes were incubated with anti-LC3A/B antibody (1:100) at 4 °C for 12 h and then incubated with TRITC-labeled goat anti-rabbit IgG H+L (1:200) at 37 °C for 1 h. For α-tubulin-FITC staining, the oocytes were incubated with anti-α-tubulin-FITC antibody (1:200) at 37 °C for 2 h. For actin staining, the oocytes were stained with phalloidin-TRITC (100 nM) at 37 °C for 1 h. Finally, oocytes were incubated with Hoechst 33,342 (10 μg/mL) at 37 °C for 15 min, then mounted on glass slides with glycerol and examined with a confocal laser scanning microscope (Zeiss LSM700 meta, Oberkochen, Germany). The fluorescence intensity was quantified and analyzed with the ImageJ 1.5 software (Bethesda, Maryland, USA).

### 5.6. Mitochondrial Function Evaluation

For mitochondria distribution detection, oocytes were loaded with MitoTracker Green fluorescent probe (100 nM) for 30 min at 37 °C and immediately fixed in 4% PFA for 30 min at RT [22]. After washing, the oocyte samples were mounted on glass slides with glycerol to analyze the abnormal rate of mitochondrial distribution by confocal microscopy.

For MMP detection, oocytes were loaded with JC-1 fluorescent probe for 30 min at 37 °C following the kit’s instructions. After washing, the oocyte samples were mounted on glass slides with PBS and examined with confocal microscopy [21]. The ratio of JC-1 red/green fluorescence intensity was analyzed with the ImageJ 1.5 software.

### 5.7. Measurement of ROS

To measure the intracellular ROS level, oocytes were loaded with 10 μM DCFHDA probe at 37 °C for 30 min. After washing, the oocyte samples were mounted on glass slides with PBS to measure the ROS levels by confocal microscopy [63]. The fluorescence intensity of the ROS signals was calculated with the ImageJ 1.5 software.

### 5.8. Annexin-V Staining

For the detection of early apoptosis, oocytes were incubated with 10 μL of Annexin-V-FITC probe in 90 μL of binding buffer at 37 °C for 20 min and immediately fixed in 4% PFA for 30 min at RT [70]. After washing, the oocyte samples were mounted on glass slides with glycerol to analyze the rate of apoptotic positive oocytes with a confocal microscope.

### 5.9. Fluorescence Intensity Analysis

The same immunostaining procedure and confocal microscope parameters were adopted for each group to optimize the acquired signals and the ImageJ 1.5 software was used to calculate fluorescence intensity. The average fluorescence intensity per unit area within the region of interest (ROI) was detected and the mean values of all measured values were used for the statistical data analyses. The fluorescence intensity of the control group was set as “1”.

### 5.10. Western Blotting

A total of 100 porcine oocytes in each group were lysed in Laemmli sample buffer and subsequently boiled at 100 °C for 10 min. The denatured proteins were separated by SDS-PAGE using 12% (*w/v*) gel and transferred to polyvinylidene fluoride (PVDF) membranes (Millipore, Billerica, MA, USA). After being blocked with 5% (*w/v*) low-fat dry milk at RT for 1 h, the membranes were incubated with primary antibodies (1:1000 for LC3A/B, BCL-2, GAPDH and α-tubulin, 1:2000 for BAX) at 4 °C for 12 h and, after five washes in TBST (Tris-buffered saline containing 0.1% (*v/v*) Tween 20), the PVDF membranes were incubated with an HRP-labeled secondary antibody (1:1000) at 37 °C for 1 h. After five washes in TBST, the protein bands were visualized with enhanced chemiluminescence solution (Biosharp, Hefei, China) and analyzed with the ImageJ 1.5 software.

### 5.11. Quantitative Real-Time PCR (qRT-PCR)

A total of 100 porcine oocytes in each group was collected to extract total RNA using TRIzol™ Reagent (Invitrogen, Carlsbad, CA, USA). The cDNA was synthesized with the PrimeScript™ RT Master Mix (TaKaRa, Tokyo, Japan) using a 20 μL reaction system (consisting of 16 μL of RNA and 4 μL of 5X Prime Script RT Master Mix) in accordance with the manufacturer’s instructions. Then, qRT-PCR was conducted with the TB Green®Premix Ex Taq™ kit (TaKaRa, Tokyo, Japan) using a 20 μL reaction system (consisting of 2 µL of cDNA, 0.4 µL of each forward and reverse primer, 10 µL of SYBR Premix Ex Taq II, 0.4 µL of ROX Reference Dye II and 6.8 µL of dd H_2_O) on a Real-time PCR instrument (Life Technologies, Gaitherburg, MD, USA). GAPDH was used as the internal control to calculate the relative mRNA expression of the target genes by the 2^−ΔΔCt^ method. The primer sequences are listed in Table 1.

### 5.12. Experimental Design

#### 5.12.1. Effects of GSP on the Meiotic Maturation of FB1-Exposed Porcine Oocytes

Previous reports have demonstrated that exposure to 30 μM FB_1_ causes meiotic defects in porcine oocytes during *IVM* [22]. In this experiment, to investigate whether GSP could alleviate the meiotic maturation defects caused by FB_1_ exposure, GSP was supplemented with IVM culture medium containing 30 µM FB_1_. The COCs were randomly allocated to five groups and 98, 98, 100, 98 and 99 oocytes were treated with 0, 30 μM FB_1_ and 30 μM FB_1_ + GSP (100 μM, 200 μM and 300 μM). After having been cultured for 44 h, the PB1 extrusion of the oocytes was examined. According to the above PB1 extrusion results, a concentration of 200 μM GSP, which significantly promoted the maturation of FB_1_-exposed oocytes, was used for further exploration.

#### 5.12.2. Effects of GSP on the Embryo Developmental Potential of FB_1_-Exposed Porcine Oocytes

To further evaluate oocytes’ developmental capacity, a total of 110, 112 and 108 MII stage oocytes in the 0, 30 μM FB_1_ and 30 μM FB_1_ + 200 μM GSP groups were activated and cultured in PZM-3 supplemented with 0.4% (*w/v*) BSA. After 48 h and 168 h of culture, the cleavage and blastocyst developmental rates were evaluated.

#### 5.12.3. Effects of GSP on the Cell Cycle Progression of FB_1_-Exposed Porcine Oocytes

To explore the reasons why GSP administration could promote porcine oocytes to extrude PB1, a total of 99, 100 and 100 oocytes were treated with 0, 30 μM FB_1_ and 30 μM FB_1_ + 200 μM GSP for 44 h. Based on the characteristics of meiotic stages as described in previous reports [63], the percentages of oocytes arrested at the GV (germinal vesicle), GVBD, MI, ATI (anaphase-telophase I) and MII stages were assessed by immunofluorescent staining.

#### 5.12.4. Effects of GSP on Spindle Assembly and Actin Distribution in FB_1_-Exposed Porcine Oocytes

To investigate the reasons why GSP administration restored cell cycle progression in FB_1_-exposed oocytes that were arrested at the GVBD stage and failed to progress to the MI stage, the cytoskeletal structure of oocytes was further examined after 28 h of culture when most oocytes were supposed to reach the MI stage. For spindle assembly evaluation, 99, 101 and 100 oocytes were treated with 0, 30 μM FB_1_ and 30 μM FB_1_ + 200 μM GSP and the spindle morphology was assessed by immunofluorescent staining of α-tubulin and DNA. For actin distribution detection, 59, 59 and 60 oocytes were treated with 0, 30 μM FB_1_ and 30 μM FB_1_ + 200 μM GSP and the actin distribution was assessed by phalloidin-TRITC staining.

#### 5.12.5. Effects of GSP on Mitochondrial Function in FB_1_-Exposed Porcine Oocytes

To investigate the protective effect of GSP on mitochondrial function in FB_1_-exposed oocytes, the mitochondrial distribution and MMP were assessed. For mitochondrial distribution detection, a total of 100, 101 and 100 oocytes were treated with 0, 30 μM FB_1_ and 30 μM FB_1_ + 200 μM GSP for 44 h, then the mitochondrial distribution in the oocytes was examined with MitoTracker Green staining. For MMP detection, a total of 100 oocytes in each group was cultured with 0, 30 μM FB_1_ and 30 μM FB_1_ + 200 μM GSP and the MMP of the oocytes was evaluated with JC-1 staining after 44 h of culture.

#### 5.12.6. Effects of GSP on Oxidative Stress in FB_1_-Exposed Porcine Oocytes

To further determine whether GSP could alleviate oxidative stress damage in FB_1_-exposed porcine oocytes, 100 oocytes in each group were treated with 0, 30 μM FB_1_ and 30 μM FB_1_ + 200 μM GSP for 44 h, ROS generation was evaluated by DCFH-DA staining. In addition, the transcriptional levels of CAT, SOD1, SOD2 and GSH-PX genes were analyzed by qRT-PCR.

#### 5.12.7. Effects of GSP on Apoptosis in FB_1_-Exposed Porcine Oocytes

To further determine whether GSP could inhibit apoptosis in FB_1_-exposed porcine oocytes, 101, 100 and 101 oocytes were treated with 0, 30 μM FB_1_ and 30 μM FB_1_ + 200 μM GSP for 44 h, then the percentage of apoptotic oocytes was evaluated by Annexin-V staining. In addition, the transcriptional level of *BAX, BCL2* and *CASPASE3* genes were analyzed by qRT-PCR and the protein expression of BAX and BCL2 were analyzed by Western blotting.

#### 5.12.8. Effects of GSP on Autophagy Levels in FB_1_-Exposed Porcine Oocytes

To further explore the impact of FB_1_ exposure and GSP cotreatment on autophagy level of oocytes, 57, 59 and 58 oocytes were treated with 0, 30 μM FB_1_ and 30 μM FB_1_ + 200 μM GSP for 44 h, then the autophagosomes were evaluated by immunofluorescent staining of LC3A/B. Additionally, the transcriptional level of *LC3, LAMP2*, *mTOR, ATG3* and *ATG5* genes were analyzed by qRT-PCR and the protein expression of LC3A/B was analyzed by Western blotting.

### 5.13. Statistical Analyses

Data from at least 3 independent replicates were analyzed for each experiment using one-way ANOVA followed by Duncan’s multiple comparisons with the GraphPad Prism 5.0 software (GraphPad Software Inc., San Diego, CA, USA). The results are presented as mean ± standard error (SE) values. *p* < 0.05 was considered statistically significant.

## Figures and Tables

**Table 1:** Primer sequences used for quantitative real-time PCR.

Gene	Primer Sequence (5′-3′)	Fragment Size (bp)
*GAPDH*	F-5’-CGTCCCTGAGACACGATGGT-3’ R-5’-GCCTTGACTGTGCCGTGGAAT-3’	194
*CAT*	F-5’-AACTGTCCCTTCCGTGCTA-3’ R-5’-CCTGGGTGACATTATCTTCG-3’	195
*GSH-PX*	F-5’-CAAGTCCTTCTACGACCTCA-3’ R-5’-GAAGCCAAGAACCACCAG-3’	210
*SOD1*	F-5’-ACCTGGGCAATGTGACTG-3’ R-5’-TCCAGCATTTCCCGTCT-3’	197
*SOD2*	F-5’-GGACAAATCTGAGCCCTAACG-3’ R-5’-CCTTGTTGAAACCGAGCC-3’	184
*BAX*	F-5’-CCAGGATCGAGCAGGGCGAAT-3’ R-5’-CACAGGGCCTTGAGCACCAGTTT-3’	285
*BCL-2*	F-5’-CAGGGACAGCGTATCAGAGC-3’ R-5’-TTGCGATCCGACTCACCAAT-3	156
*CASPASE-3*	F-5’-GAACTCTAACTGGCAAACCCAA-3’ R-5’-GCATACAAGAAGTCTGCCTCAA-3’	142
*LC3*	F-5’-CCGAACCTTCGAACAGAGAG-3’	206
	R-5’-AGGCTTGGTTAGCATTGAGC-3’	
*LAMP2*	F-5’-GCTTTTGCAGCGTTGTGG-3’	169
	R-5’-GACGAGGCAGAGCATAAGGAG-3’	
*mTOR*	F-5’-GCACAAGGACGGATTCCTAC-3’	248
	R-5’-CACTTGCGTTGGGAGATC-3’	
*ATG3*	F-5’-CACGACTATGGTTGTTTGGCTATG-3’	127
	R-5’-GGTGGAAGGTGAGGGTGATTT-3’	
*ATG5*	F-5’-CCTGAAGATGGGGAAAGAAAGA-3’	140
	R-5’-TCTGTTGGTTGCGGGATG-3’	

## Data Availability

Data available in a publicly accessible repository.
